# Isolation of Kyasanur Forest Disease Virus from Febrile Patient, Yunnan, China

**DOI:** 10.3201/eid1502.080979

**Published:** 2009-02

**Authors:** Jinglin Wang, Hailin Zhang, Shihong Fu, Huanyu Wang, Daxin Ni, Roger Nasci, Qing Tang, Guodong Liang

**Affiliations:** State Key Laboratory for Infectious Disease Prevention and Control, Beijing, China (J. Wang, S. Fu, H. Wang, D. Ni, Q. Tang, G. Liang); Yunnan Institute of Endemic Disease Control and Prevention, Dali, China (J. Wang, H. Zhang); Centers for Disease Control and Prevention, Fort Collins, Colorado, USA (R. Nasci)

**Keywords:** Kyasanur Forest disease virus (KFDV*)*, Alkhurma virus, Nanjianyin virus

## Abstract

We recently determined that Nanjianyin virus, isolated from serum of a patient in Yunnan Province, China, in 1989, is a type of Kyasanur Forest disease virus. Results of a 1987–1990 seroepidemiologic investigation in Yunnan Province had shown that residents of the Hengduan Mountain region had been infected with Nanjianyin virus.

Kyasanur Forest disease (KFD) virus, a member of the tick-borne encephalitis virus serocomplex of the genus *Flavivirus*, family *Flaviviridae*, can cause fever, hemorrhage, and encephalitis and has a 3%–5% case-fatality ratio (*1*). KFD was discovered in 1957 in the Mysore forest region of south India, where 400–500 persons per year were infected with the virus (*2*,*3*). KFD virus has been found only in monkeys, humans, and *Haemaphysalis spinigera* ticks in the KFD-epidemic region of south India (*4*), although a variant of KFD virus, Alkhurma virus, was isolated recently in Saudi Arabia (*5*). In this study, we determined that the gene sequence of a Nanjianyin virus isolate obtained from a febrile patient is highly homologous to that of KFD virus. The Nanjianyin virus was isolated in 1989 from the serum of a 38-year-old woman from the Hengduan Mountain region of Yunnan Province, People’s Republic of China, where a previous serosurvey demonstrated that KFD exposure had occurred (Figure 1).

**Figure 1:**
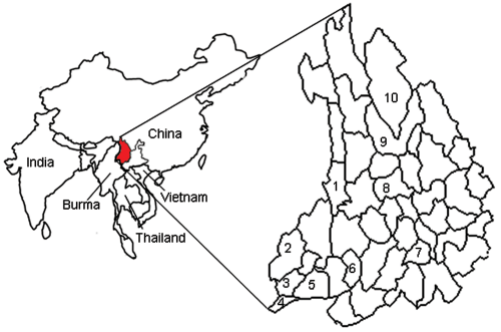
Counties in the Hengduan Mountain region of Yunnan Province where Kyasanur Forest disease virus antibody has been detected. 1, Lushuii County, antibody found in 31.6% of humans, 25.5% of birds, and 15.4% of rodents; 2, Yingjiang County, antibody found in 46.7% of humans; 3, Longchuang County, antibody found in 6.4% of humans; 4, Ruili County, antibody found in 7.7% of humans; 5, Mangshi County, antibody found in 32.5% of humans; 6, Shidan County, antibody found in 6.3% of humans; 7, Nanjian County, county in which Nanjianyin virus was found in 1989; 8, Eryuan County, antibody found in 4.9% of birds; 9, Lijiang County, antibody found in 0.7% of humans; 10, Shangri-La County, antibody found in 8.5% of humans.

## The Study

In tests conducted shortly after isolation of Nanjianyin virus in 1989, the virus caused a typical cytopathic effect within 4 days after its injection in BHK-21 cells, killed 100% of 3-day-old mice within 2.5 days after their intracerebral inoculation with a 25-μL culture supernatant, and killed 100% of 50-day-old adult mice within 11–13 days of their intraperitoneal inoculation with a 30-μL culture supernatant. Hemagglutination inhibition test results showing a cross-reaction between Nanjinayin virus and a Japanese encephalitis virus antibody indicated that Nanjianyin virus belonged to the genus *Flavivirus.* No further tests to classify Nanjianyin virus were performed at the time it was isolated. The virus was preserved by lyophilization and stored at –30°C.

Recently, we used molecular methods to determine that Nanjianyin virus is a variant of KFD virus. After reconstituting the lyophilized virus in a BioSafety Level 3 biosafety cabinet, we suspended the sample in 0.5 mL minimum essential media (Gibcol BRL, Gaithersburg, MD, USA) (pH 7.4) and then centrifuged it for 5 min at 6,000× *g*. We then extracted the total RNA from 140 µL of supernatant by using the QIAamp Viral RNA Mini Kit (QIAGEN, Valencia, CA, USA) in accordance with the manufacturer’s protocol and produced the first strands of cDNA by using Ready-To-Go You-Prime First-Strand Beads (Amersham Pharmacia Biotech, Piscatawy, NJ, USA) as described in the manual accompanying the kit. We used *Flavivirus* genus-specific primers (*6*) to perform reverse transcription-PCR amplification using viral genomic RNA as a template and determined the nucleotide sequence of the virus from the amplified cDNA fragment. Results of nucleotide sequence analysis by BLAST (http://blast.ncbi.nlm.nih.gov/Blast.cgi) showed that the nucleotide DNA sequence of Nanjianyin virus was 99% homologous to that of KFD virus (prototype KFDV *Itp9605,* GenBank accession no. AY323490).

To complete the sequence determination of the PrM-E genes, we designed 3 pairs of primers to amplify them. Using information from a previous study (*6*), we also designed an additional primer pair to amplify the nonstructural protein (NS5) gene (Table).

**Table:** Primers used to sequence the PrM-E and NS5 genes of Nanjianyin virus*

Primers	Primers sequence (5′ → 3′)
PrM-E gene primers	
KFD1F(105-124)	CGGACTGGTATTGATGCG
KFD1R(1357-1340)	TCTTCTCGGACTGCGTTG
KFD2F(1100-1117)	ACCAGGCGAGCACAGTCT
KFD2R(1952-1935)	CCTCCTCCAGTTGTTTCCA
KFD3F(1652-1673)	GAGTGCCCGTGGCTAACATAGA
KFD3R(2832-2812)	CTTGGTCCTCATTCCCATCCC
NS5 gene primers	
FU1PM (8908-8933)	TACAACATGATGGGVAARAGWGARAA
cFD3 (9961-9983)	AGCATGTCTTCCGTGGTCATCCA

*NS, nonstructural protein.

**Figure 2:**
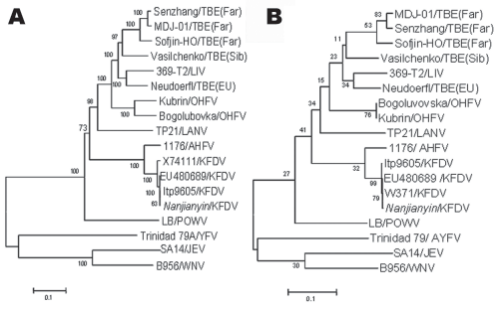
Phylogenetic analysis of the PrM-E (A) and nonstructural protein 5 (B) gene sequences of Nanjianyin virus isolated from Yunnan Province, China. Phylogenetic analyses were performed by the neighbor-joining method with MEGA version 3.1 software (www.megasoftware.net). Bootstrap probabilities of each node were calculated with 500 replicates. Scale bars indicate number of nucleotide substitutions per site. Abbreviations and GenBank accession numbers are as follows: tick-borne encephalitis virus (TBEV), TBEV (Far), strain MDJ-01(AY217093); TBEV (Far), strain Senzhang (AY182009); TBEV (Far), strain Sofjin-HO (AB062064); TBEV (Sib), strain Vasilchenko (AF069066); louping iII virus (LIV), strain 369-T2 (Y07863); TBEV (EU), strain Neudoerfl (U27495); Omsk hemorrhagic fever virus (OHFV), strain Bogoluvovska (AY193805); OHFV, strain Kubrin (AY438626); Langat virus (LANV), strain TP21 (AF253419); Alkhurma hemorrhagic fever virus (AHFV), strain 1176 ( AF331718); Kyasanur Forest disease virus (KFDV), strain unknown (PrM-E, X74111); KFDV, strain unknown (EU480689); KFDV, strain Itp6905 (AY323490); KFDV, strain W371 (NS5, AF013385); KFDV, strain Nanjianyin (PrM-E, EU918175; NS5, EU918174); Powassan virus (POWV), strain LB ( L06436); yellow fever virus (YFV), strain Trinidad 79 (AF094612); Japanese encephalitis virus (JEV), strain SA14 (D90194); West Nile virus (WNV), strain B956 (AY532665).

Results of sequence alignment and homology analysis performed with MegAlign software of DNASTAR (Madison, WI, USA) showed that the 654-bp PrM gene of Nanjianyin virus was 99.6% identical to that of KFD virus (Itp9605 strain), 99.4% identical to that of KFD virus (EU480489), 98.2% identical to that of KFD virus (X74111), but only 90.4% identical to that of Alkhurma hemorrhagic fever (AFH) virus (1176 strain), and only 57.2% to 64.3% identical to the 654-bp PrM genes of other tick-borne encephalitis complex viruses such as Omsk hemorrhagic fever virus (Kubrin strain), tick-borne encephalitis virus (Sengzhang strain), Powassan virus (LB strain), and Langat virus (TP21 strain). The 1487-bp E gene nucleotide sequence of Nanjianyin virus was 99.8% identical to that of KFD virus (Itp9605 strain), 99.8% identical to that of KFD virus (EU480489), 98.5.0% identical to that of KFD virus (X74111), 91.9% identical to that of AFH virus (1176 strain), and <72% identical to that of other tick-borne encephalitis complex viruses. The nucleotide sequence of the 1,000-bp NS5 gene of Nanjianyin virus was 99.6%, 99.7%, and 99.7% homologous to that of KFD virus (Itp9605 strain), KFD virus (W371), and KFD virus (EU480489), respectively; 92.3% homologous to that of AFH virus isolate 1176; and <77.6% homologous to the 1,000-bp NS5 gene of other tick-borne encephalitis complex viruses. Results of homology analyses thus demonstrated that Nanjianyin virus belongs to the KFD virus clade, and results of phylogenetic analyses conducted with 2,142 nt of the PrM-E gene and 1,000 nt of the NS5 gene suggested that Nanjianyin virus and KFD virus are in the same genetic cluster (Figure 2).

## Conclusions

Results of a serosurvey of tick-borne viruses conducted from 1987 through 1990 in Yunnan Province (*7*) showed that 169 (19.5%) of 867 healthy residents of western Yunnan Province (in Lushui, Shidian, Yingjiang, Mangshi, Ruili, and Longchuan counties) and 6 (3.7%) of 161 healthy residents of northwestern Yunnan Province (in Lijiang and Diqin counties) carried antibodies against KFD virus. KFD antibodies also were detected in the serum of patients with fever in Lushui County (*7*,*8*) and in the serum of resident birds, migratory birds, rodents, and rhesus monkeys (*Macaca mulatta*) in the Hengduan Mountain region (Lushui and Eryuan counties) (*7*,*9*). These results indicate that humans and animals in the Hengduan Mountain region of Yunnan Province have been infected with KFD virus since the 1980s. Although detailed information about the movement of the woman infected with Nanjianyin virus in 1989 is not available, residents of the Hengduan Mountain region at that time seldom traveled far, so she probably was exposed there.

Results of epidemiologic and virologic investigations suggest that migratory birds play a key role in the spread of arboviruses (*10*,*11*). Migratory birds frequently pass through Yunnan Province during their migration from south India and the Indian Ocean islands to Mongolia and Siberia. The areas adjacent to Hengduan Mountain in Yunnan Province and India also provide a suitable habitat for *Haemaphysalis spinigera,* which is the vector for KFD virus in the region (*12*,*13*). Our results, combined with those in previous seroprevalence reports of KFD virus in humans and birds (*6*,*7*), indicate that KFD virus likely was carried to the region by these migratory birds and their parasitic ticks. KFD antibodies have been detected in residents of north and northeast India, and the KFD seropositive rate is especially high among residents of India’s Andaman Islands and Nicobar Islands (*14*). KFD antibodies also were detected in both human and bird serum in the Chinese districts of Guangdong, Guangxi, Guizhou, Hubei, Henan, Xinjiang, and Qinghai in 1983 (*15*).

In summary, we found that Nanjianyin virus, first isolated in the Hengduan Mountain region of Yunnan Province, is a variant of KFD virus. This finding confirms that infection with KFD virus has previously occurred in the region and justifies enhanced surveillance for KFD among febrile patients in the Hengduan Mountain region.
